# The Returning Troops

**Published:** 1900-11-10

**Authors:** 


### THE RETURNING TROOPS.

Lord Roberts writes from the headquarters of the Army in South Africa, dated Pretoria, September 30th, 1900: "Will you kindly allow me, through the medium of your paper, to make an appeal to my countrymen and women upon a subject I have very much at heart, and which has been occupying my thoughts for some time past. All classes in the United Kingdom have shown such a keen interest in the Army serving in South Africa, and have been so munificent in their efforts to supply every need of that Army, that I feel sure they must be eagerly looking forward to its return, and to giving our brave soldiers and sailors the hearty welcome they so well deserve when they get back to their native land. It is about the character of this welcome, and the effect it may have on the reputations of the troops whom I have been so proud to command, that I am anxious, and that I venture to express an opinion. My sincere hope is that the welcome may not take the form of 'treating' the men to stimulants in public-houses or in the streets, and thus lead them into excesses which must tend to degrade those whom the nation delights to honour, and to lower the 'Soldier of the Queen' in the eyes of the world—that world which has watched with undisguised admiration the grand work they have performed for their Sovereign and their country. From the very kindness of their hearts, their innate politeness, and their gratitude for the welcome accorded them, it will be difficult for the men to refuse what is offered to them by their too generous friends. I, therefore, beg earnestly that the British public will refrain from tempting my gallant comrades, but will rather aid them to uphold the splendid reputation they have won for the Imperial Army. I am very proud that I am able to record with the most absolute truth, that the conduct of this army from first to last has been exemplary. Not one single case of serious crime has been brought to my notice—indeed, nothing that deserves the name of *crime*. There has been no necessity for appeals or orders to the men to behave properly. I have trusted implicitly to their own soldierly feeling and good sense, and I have not trusted in vain. They bore themselves like heroes on the battlefield, and like gentlemen on all other occasions. Most malicious falsehoods were spread abroad by the authorities in the Orange Free State and the Transvaal as to the brutality of Great Britain's soldiers, and as to the manner in which the women and children might expect to be treated. We found, on first entering towns and villages, doors closed and shops shut up, while only English-born people were to be seen in the streets. But very shortly all this was changed. Doors were left open, shutters were taken down, and people of all nationalities moved freely about, in the full assurance that they had nothing to fear from "the man in khaki," no matter how battered and war-stained his appearance. This testimony will, I feel sure, be very gratifying to the people of Great Britain, and of that Greater Britain whose sons have shared to the fullest extent in the suffering as well as the glory of the war, and who have helped so materially to bring it to a successful close. I know how keen my fellow-subjects will be to show their appreciation of the upright and honourable bearing as well as the gallantry of our sailors and soldiers, and I would entreat them, in return for all these grand men have done for them, to abstain from any action that might bring the smallest discredit upon those who have so worthily upheld the credit of their country. I am induced to make this appeal from having read, with great regret, that when our troops were leaving England, and passing through the streets of London, their injudicious friends pressed liquor upon them and shoved bottles of spirits into their hands and pockets—a mode of 'speeding the parting' friend which resulted in some very distressing and discreditable scenes. I fervently hope there may be no such scenes to mar the brightness of the Welcome Home."

